# A Critical Appraisal of the Hippocampal Subfield Segmentation Package in FreeSurfer

**DOI:** 10.3389/fnagi.2014.00261

**Published:** 2014-09-25

**Authors:** Laura E. M. Wisse, Geert Jan Biessels, Mirjam I. Geerlings

**Affiliations:** ^1^Julius Center for Health Sciences and Primary Care, University Medical Center, Utrecht, Netherlands; ^2^Department of Neurology, Brain Center Rudolf Magnus, University Medical Center, Utrecht, Netherlands

**Keywords:** hippocampal subfields, FreeSurfer, automated segmentation, hippocampus, Alzheimer

In the last decade, the *in vivo* assessment of hippocampal subfields has received increasing attention because of the differential role of hippocampal subfields in several neuropsychiatric diseases (Geuze et al., 2005). Several manual segmentation protocols have been developed for 3–7 T MRI (Mueller et al., 2007; Van Leemput et al., 2008; La Joie et al., 2010; Wisse et al., 2012), some of which are automated (Van Leemput et al., 2008; Yushkevich et al., 2009). One of these automated protocols (Van Leemput et al., 2008, 2009) has recently been implemented in FreeSurfer (Fischl, 2012), a freely available easy-to-use set of automated brain MRI analysis tools. This has made hippocampal subfield segmentation available to everyone with 1.5–3 T MRI data and the method is being used in an increasing number of studies (Teicher et al., 2012; Li et al., 2013; Pereira et al., 2014).

In this commentary, we express our concern with the hippocampal subfield segmentation package in FreeSurfer. In particular, we address issues concerning (1) image acquisition, (2) the parcelation scheme, and (3) validation of this automated segmentation.

The first concern with the hippocampal subfield segmentation package in FreeSurfer is that it requires low resolution (1 mm^3^) T1 images (whole-brain). Most other manual or automated segmentation methods are developed for high-resolution T2 images (in-plane: 0.20–0.70 mm^2^, often with partial-brain coverage) (Mueller et al., 2007; Kerchner et al., 2010; La Joie et al., 2010; Wisse et al., 2012). On high-resolution T2 images, contrast between white and gray matter is sufficient to visualize the white matter bands between the dentate gyrus and the cornu ammonis (CA) that are generally used as a boundary between these subfields. The low resolution T1 images on which the FreeSurfer segmentation is applied do not contain this amount of detail. See Figure 1 for a comparison of low resolution T1 and high-resolution T2 images.

**Figure 1:**
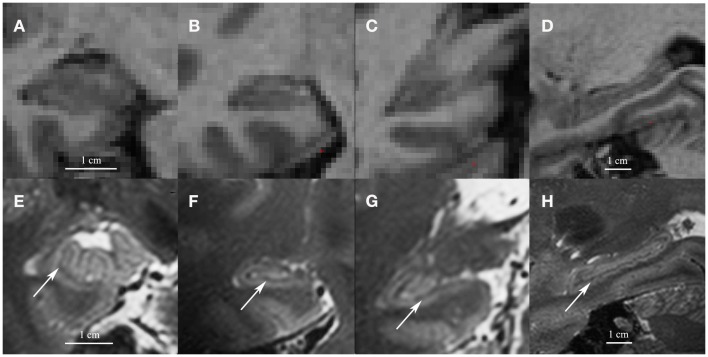
**Coronal images of the head (A), body (B) and tail (C) and a sagittal cross-section of the hippocampus (D) on low resolution 1mm^3^ T1 1.5 T images (A–D) and on high resolution 0.7 mm^3^ T2 7 T images (E–H)**. Note the white matter bands between the dentate gyrus and cornu ammonis on the high resolution T2 images (indicated by arrows). Although we show high resolution 7 T T2 images here, the white matter bands between the dentate gyrus and the cornu ammonis can also be visualized on high resolution T2 3–4 T images (Mueller et al., 2007; La Joie et al., 2010; Winterburn et al., 2013).

The second concern is the parcelation scheme used for the FreeSurfer segmentation, which is based on the subfield distribution in one coronal section in the body of the hippocampus (Van Leemput et al., 2008, 2009) and then used to segment subfields along the complete long axis of the hippocampus. However, the presence and position of the subfields differ along the long axis (Duvernoy et al., 2005; Mai et al., 2008; Insausti and Amaral, 2012). Consequently, the locations of the boundaries between subfields in this segmentation protocol are in mismatch with the anatomical atlases in a large part of the long axis. For example, in FreeSurfer, the dentate gyrus is segmented from the anterior pole of the hippocampus, while it only becomes visible 6 mm after the anterior pole of the hippocampus (Insausti and Amaral, 2012). Several segmentation methods exist also for T2 images, manual (La Joie et al., 2010; Wisse et al., 2012) as well as automated (Yushkevich et al., 2009). Because of the complex anatomy of the hippocampal head and tail, these methods either limit the segmentation of subfields to the hippocampal body (Mueller et al., 2007; Yushkevich et al., 2009) or developed a separate segmentation scheme for the head and/or tail (La Joie et al., 2010; Wisse et al., 2012; Winterburn et al., 2013).

As a consequence of the placement of the subfield boundaries in FreeSurfer, large parts of subfields are assigned to neighboring subfields. For example, large parts of CA1 are included in the subiculum and CA2&3. This generates volume estimates that are in contrast with anatomical studies. In studies using the FreeSurfer segmentation package (e.g., Teicher et al., 2012; Boen et al., 2014), CA2&3 is the largest subfield, while CA1 is the smallest. According to anatomical studies, CA1 is the largest and CA2&3 is the smallest subfield (Simic et al., 1997; Rossler et al., 2002). In general, subfield boundaries are difficult to discern *in vivo* and part of subfields are counted toward neighboring subfields in all segmentation protocols. However, other manual or automated methods generate subfield estimates that are more in line with those of anatomical studies (e.g., Wisse et al., 2012; Winterburn et al., 2013). See Table S1 in Supplementary Material for a comparison of subfield volumes and their percentage distribution within the hippocampus according to several segmentation protocols.

Studies using this FreeSurfer segmentation package to investigate hippocampal subfield volumes in mild cognitive impairment (MCI) and Alzheimer disease (AD) reported results that differ from anatomical studies. Several studies using the FreeSurfer package reported that MCI and AD were mainly related to CA2&3 atrophy (Hanseeuw et al., 2011; Lim et al., 2012). These latter results stand in contrast to the anatomical studies that reported the greatest atrophy in CA1 (Simic et al., 1997; Rossler et al., 2002). Perhaps, CA2&3 atrophy in MCI or AD in studies using FreeSurfer actually represents CA1 atrophy, as a large part of CA1 is counted toward CA2&3 in FreeSurfer. Studies using other manual or automated segmentation methods reported subfield atrophy in AD that more closely matched the results of anatomical studies (Mueller and Weiner, 2009; Pluta et al., 2012; La Joie et al., 2013).

A third concern is that the automated segmentation in FreeSurfer was developed on high-resolution (0.19 mm × 0.19 mm × 0.80 mm) 3 T images and is now applied on low resolution (1 mm^3^) images. To the best of our knowledge, the protocol was not validated against a manual segmentation on these lower resolutions 1.5–3 T MR images (see also Lim et al., 2012; Pluta et al., 2012). Moreover, it should be noted that the intra-rater reliability of the manual segmentation used for the FreeSurfer package was based on repeated segmentation of two coronal slices rather than on segmentation of the complete long axis of the hippocampus (Van Leemput et al., 2009).

In conclusion, though FreeSurfer provides a useful, broad set of automated brain MRI analysis tools, we have concerns about the current package for automated hippocampal subfield segmentation. The boundaries of the parcelation scheme are in mismatch with known anatomical boundaries. This will impact the reliability of studies using FreeSurfer to investigate subfield atrophy in neuropsychiatric diseases.

## Author Contributions

Laura E. M. Wisse: study concept and design, wrote the manuscript, final approval and agreement to be accountable for all aspects of the work. Mirjam I. Geerlings: study concept and design, critical revision of the manuscript for important intellectual content, final approval and agreement to be accountable for all aspects of the work. Geert Jan Biessels: study concept and design, critical revision of the manuscript for important intellectual content, final approval and agreement to be accountable for all aspects of the work.

## Conflict of Interest Statement

The authors declare that the research was conducted in the absence of any commercial or financial relationships that could be construed as a potential conflict of interest.

## Supplementary Material

The Supplementary Material for this article can be found online at http://www.frontiersin.org/Journal/10.3389/fnagi.2014.00261/full

## Abbreviations

AD, Alzheimer disease; CA, cornu ammonis; MCI, mild cognitive impairment.
