# Supplementary material for: A Critical Appraisal of the Hippocampal Subfield Segmentation Package in FreeSurfer
Source: Front Aging Neurosci. 2014 Sep 25;6:261. doi: 10.3389/fnagi.2014.00261 (PMC4174865; doi:10.3389/fnagi.2014.00261)
Supplement: Supplementary file 1 [file Table1.DOCX]

***Supplementary Material***

**A critical appraisal of the hippocampal subfield segmentation package in FreeSurfer**

**Laura E.M. Wisse^1,2^, Geert Jan Biessels^2^, Mirjam I. Geerlings^1^***

^1^Julius Center for Health Sciences and Primary Care, University Medical Center, Utrecht, The Netherlands
^2^Department of Neurology, Brain Center Rudolf Magnus, University Medical Center, Utrecht, The Netherlands

*** Correspondence:** M.I. Geerlings, Julius Center for Health Sciences and Primary Care, University Medical Center Utrecht, Stratenum 6.131 PO BOX 85500, Utrecht, 3508 GA, The Netherlands.
m.geerlings@umcutrecht.nl

1. **Supplementary Figures and Tables**

## Supplementary Tables

**Supplementary Table 1. A comparison subfield volumes and their percentage distribution within the hippocampus according to several segmentation protocols**

|  | **Anatomical Studies** | | **Studies using the FreeSurfer segmentation** | | **Other segmentation protocols using high resolution T2 images*** | |
| --- | --- | --- | --- | --- | --- | --- |
|  | **Rossler et al. (2002)^1^** | **Simic et al. (1997)^2^** | **Boen et al. (2014)^3^** | **Teicher et al. (2012)^4^** | **Winterburn et al. (2013)^5^** | **Wisse et al. (2012)^6^** |
| **Subiculum** Volume (mL) % of hippocampal volume | 0.43 30 | 0.45 29 | 1.04 31 | 0.96 37 | 0.39 14 | 0.80 22 |
| **CA1** Volume (mL) % of hippocampal volume | 0.54 37 | 0.64 42 | 0.32 9 | 0.28 11 | 0.86 31 | 1.71 46 |
| **CA2&3** Volume (mL) % of hippocampal volume | 0.15 10 | 0.14 9 | 0.99 29 | 0.85 32 | 0.21 8 | 0.25 7 |
| **DG(&CA4)** Volume (mL) % of hippocampal volume | - - | - - | 0.55 16 | 0.47 18 | 0.62 22 | 0.93 25 |

*We chose two protocols that segmented the hippocampus in the full-length and separated CA2 and 3 from the dentate gyrus and could thus be compared to the FreeSurfer segmentation.

CA=cornu ammonis; DG=dentate gyrus. ^1^Data derived from table 1, 'Stage I'; ^2^Data derived from table 2, 'Normal'; ^3^Data derived from table 2, 'Control', average of left and right, subiculum is the sum of presubiculum and subiculum; ^4^Data derived from table 4, ACE=0), average of left and right, subiculum is the sum of presubiculum and subiculum; ^5^Data derived from table 3, SR/SL/SM has not been taken into account; ^6^Data derived from table 1.

1. **References**

Boen, E., Westlye, L.T., Elvsashagen, T., Hummelen, B., Hol, P.K., Boye, B., Andersson, S., Karterud, S., and Malt, U.F. (2014). Smaller stress-sensitive hippocampal subfields in women with borderline personality disorder without posttraumatic stress disorder*. J. Psychiatry Neurosci.* 39, 127-134

Rossler, M., Zarski, R., Bohl, J., and Ohm, T.G. (2002). Stage-dependent and sector-specific neuronal loss in hippocampus during Alzheimer's disease*. Acta Neuropathol.* 103, 363-369

Simic, G., Kostovic, I., Winblad, B., and Bogdanovic, N. (1997). Volume and number of neurons of the human hippocampal formation in normal aging and Alzheimer's disease*. J. Comp. Neurol.* 379, 482-494

Teicher, M.H., Anderson, C.M., and Polcari, A. (2012). Childhood maltreatment is associated with reduced volume in the hippocampal subfields CA3, dentate gyrus, and subiculum*. Proc. Natl. Acad. Sci. U. S. A.* 109, E563-E572

Winterburn, J.L., Pruessner, J.C., Chavez, S., Schira, M.M., Lobaugh, N.J., Voineskos, A.N., and Chakravarty, M.M. (2013). A novel in vivo atlas of human hippocampal subfields using high-resolution 3 T magnetic resonance imaging*. Neuroimage* 74, 254-265

Wisse, L.E.M., Gerritsen, L., Zwanenburg, J.J., Kuijf, H.J., Luijten, P.R., Biessels, G.J., and Geerlings, M.I. (2012). Subfields of the hippocampal formation at 7 T MRI: in vivo volumetric assessment*. Neuroimage* 61, 1043-1049
